# Case report: Systemic T-DXd and bevacizumab plus local therapy prolongs survival in breast cancer leptomeningeal metastasis: a case series and literature review

**DOI:** 10.3389/fonc.2026.1961848

**Published:** 2026-09-24

**Authors:** Junmei Zhang, Xuezheng Wang, Tiezhu Li, Luyao Yang, Yan Xue

**Affiliations:** 1 Department of Medical Oncology, Xi’an International Medical Center Hospital, Xi’an, Shaanxi, China; 2 Mailman School of Public Health, Columbia University Irving Medical Center, New York, NY, United States

**Keywords:** bevacizumab, breast cancer, intrathecal injection, leptomeningeal metastases (LM), trastuzumab deruxtecan (T-DXd), whole-brain radiotherapy (WBRT)

## Abstract

Leptomeningeal metastasis from breast cancer has a low incidence but an extremely poor prognosis. With the development of antitumor therapies, the survival of patients with metastatic breast cancer has been significantly prolonged. However, due to the presence of the blood–brain barrier, intracranial metastasis has become increasingly prominent, and the incidence of leptomeningeal metastasis is gradually rising, warranting greater attention and improvement. Currently, there is a lack of effective treatment options for leptomeningeal metastasis, and no unified diagnostic or therapeutic standards exist. This case series reports seven breast cancer patients with leptomeningeal metastasis who received trastuzumab deruxtecan combined with whole-brain radiotherapy; four of them also received bevacizumab and intrathecal chemotherapy. In these four patients, the addition of bevacizumab and intrathecal therapy was associated with rapid alleviation of central nervous system symptoms and a numerically longer overall survival (9.0–20.2 months) compared with historical data. No severe or fatal adverse events were observed. Additionally, through a review of the literature, we summarize the current status, diagnosis, treatment, prognosis, and potential future directions of leptomeningeal metastasis from breast cancer, aiming to provide reference for the management of these patients.

## Introduction

Leptomeningeal metastasis (LM) refers to the spread of malignant tumors to the leptomeninges and subarachnoid space. The most common solid tumors causing LM are malignant melanoma, lung cancer, and breast cancer (1). In breast cancer patients with LM, the presence of invasive lobular carcinoma or triple-negative breast cancer (TNBC) confers a relatively higher risk (2). Among breast cancer patients with LM, lobular carcinoma histology accounts for approximately 17%–28% of cases (3, 4). Regarding molecular subtypes of LM, TNBC accounts for approximately 21%–40.5% (3, 5, 6). In addition, human epidermal growth factor receptor 2 (HER2)-positive breast cancer is also a molecular subtype prone to LM, accounting for approximately 14%–29% of cases (2, 6–10).

The prognosis for breast cancer patients with LM is extremely poor. Without antitumor therapy, the median overall survival (mOS) is only 4–6 weeks (11). Even with various treatment approaches attempted in the past, the mOS has only reached 1.75–4.5 months (12). Systemic drug therapy is generally indispensable; however, due to the presence of the blood–brain barrier and the blood–cerebrospinal fluid barrier, the drug concentration penetrating into the cerebrospinal fluid is insufficient. Moreover, drugs are readily cleared by active efflux transporters, resulting in limited efficacy of conventional therapies. In recent years, the emergence of novel targeted agents, represented by small-molecule tyrosine kinase inhibitors (TKIs) and antibody–drug conjugates (ADCs), has improved the efficacy of systemic therapy against intracranial tumors. Bevacizumab, an anti-vascular endothelial growth factor (VEGF) monoclonal antibody, can rapidly alleviate cerebral edema and improve antitumor drug delivery by reducing tumor vascular permeability and decreasing interstitial pressure, thereby exerting a synergistic effect (13, 14). In addition to systemic therapy, combined multimodal approaches incorporating local treatments are being actively explored. Intrathecal injection can significantly increase drug concentrations in the cerebrospinal fluid and alleviate central nervous system symptoms. Cranial radiotherapy can disrupt the blood–brain barrier to a certain extent, facilitating subsequent drug entry into the cerebrospinal fluid.

Currently, there is still a lack of standardized, unified, and effective diagnostic and therapeutic recommendations for breast cancer patients with LM. In this article, we report our preliminary real-world experience with a multimodal treatment approach in seven breast cancer patients with LM. These patients received trastuzumab deruxtecan (T-DXd)-based systemic therapy combined with whole-brain radiotherapy (WBRT), with or without bevacizumab and intrathecal injection. Our primary purpose is to describe the clinical outcomes observed in this small cohort and to generate hypotheses for future prospective studies on combination strategies for LM.

## Methods

This was a retrospective case series, not a prospective interventional clinical trial. All treatments were administered at the discretion of the treating physicians based on real-world clinical practice and patient condition.

All patients received intravenous T-DXd at 5.4 mg/kg every three weeks, combined with WBRT at 40 Gy in 20 fractions. Among them, four patients also received intravenous bevacizumab at 7.5 mg/kg every three weeks and weekly intrathecal injections of methotrexate (10 mg) plus cytarabine (50 mg). The combination of T-DXd and bevacizumab was chosen based on emerging evidence suggesting potential synergistic effects. All patients provided informed consent for off-label use prior to treatment initiation. Written informed consent for publication of case details and images was obtained from the patients’ legal guardians/next of kin. This study was reviewed and approved by the Ethics Committee of Xi’an International Medical Center Hospital (Approval No. GJYX-KY-2026-055).

Leptomeningeal metastasis was diagnosed based on a combination of clinical symptoms, contrast-enhanced cranial magnetic resonance imaging (MRI) findings, and cerebrospinal fluid (CSF) cytology. This study primarily evaluated the response of leptomeningeal lesions. For leptomeningeal lesions, the RANO-LM criteria were applied based on changes in meningeal enhancement on contrast-enhanced MRI. Best response was determined by the investigator based on integrated imaging and clinical assessment. For patients who died of disease progression before follow-up imaging could be performed, the best response was recorded as PD based on clinical deterioration in conjunction with the treating physician’s assessment.

## Case 1

A 76-year-old female was diagnosed with right-sided breast cancer in July 2015. Immunohistochemistry revealed estrogen receptor (ER)-negative, progesterone receptor (PR)-negative, HER2 3+, and Ki67 positivity of 25%, consistent with HER2-positive breast cancer. The patient’s initial diagnosis was stage IV (T4N2M1), with right axillary lymph node and multiple bone metastases. Subsequently, she developed metastases to the liver, pericardium, pleura, and chest wall. During the advanced stage, the patient received five lines of antitumor therapy: epirubicin plus cyclophosphamide sequentially followed by docetaxel (initial anti-HER2 therapy was not administered due to financial constraints), trastuzumab plus tegafur–gimeracil–oteracil, pyrotinib plus cisplatin, trastuzumab plus pertuzumab plus eribulin, and inetetamab plus pyrotinib plus capecitabine.

In February 2022, the patient developed symptoms including dizziness, vomiting, gait instability, confusion, and incoherent speech. Cranial MRI revealed multiple abnormal enhancing signals in the sulci of the bilateral frontal, parietal, temporal, occipital lobes, and the cerebellar vermis, suggestive of leptomeningeal metastasis. Lumbar puncture pathology demonstrated the presence of cancer cells in the CSF. In February 2022, the patient received two cycles of intravenous T-DXd, combined with WBRT. After treatment, the patient’s dizziness and vomiting improved slightly, but the psychiatric abnormalities did not show significant improvement, and her physical condition gradually deteriorated. The patient died on April 7, 2022. The progression-free survival (PFS) and OS were only 2.1 months.

## Case 2

A 48-year-old female was diagnosed with left-sided breast cancer in August 2017. Immunohistochemistry revealed ER-negative, PR-negative, HER2 1+, and Ki67 positivity of 10%, consistent with TNBC. The patient’s initial diagnosis was stage IIB (T2N1M0), with left axillary lymph node metastasis. Genetic testing revealed a germline *BRCA2* pathogenic mutation. The patient first received neoadjuvant chemotherapy with epirubicin plus cyclophosphamide sequentially followed by docetaxel. On November 9, 2017, she underwent modified radical mastectomy of the left breast. In November 2018, she began to develop distant metastases, involving the ovaries, pleura, and bones. During the advanced stage, the patient received two lines of antitumor therapy: olaparib and eribulin.

In May 2022, the patient developed severe headache, nausea, vomiting, blurred vision, and epileptic seizures. MRI revealed thickening and marked enhancement of the meninges in the right frontal and temporal lobes, suggestive of LM (**Figure 1A**). Lumbar puncture pathology demonstrated a large number of cancer cells in the CSF (**Figure 1C**). In May 2022, the patient received intravenous T-DXd plus bevacizumab, combined with WBRT, and weekly intrathecal injections of methotrexate plus cytarabine. After the initiation of intrathecal therapy, the patient’s central nervous system symptoms improved rapidly, and basically resolved after three intrathecal injections. In July 2022, follow-up cranial MRI revealed that the previous thickening and enhancement of the meninges in the right frontal and temporal lobes had resolved (**Figure 1B**). Cancer cells in the cerebrospinal fluid were significantly reduced compared with those before treatment (**Figure 1D**). In October 2022, the patient again developed obvious headache, nausea, and vomiting, and lumbar puncture again revealed a large number of cancer cells; the disease was considered to have progressed, and other treatment regimens were switched. The patient died on March 28, 2023. The patient’s PFS was 5.1 months, and OS was 9.9 months.

**Figure 1:**
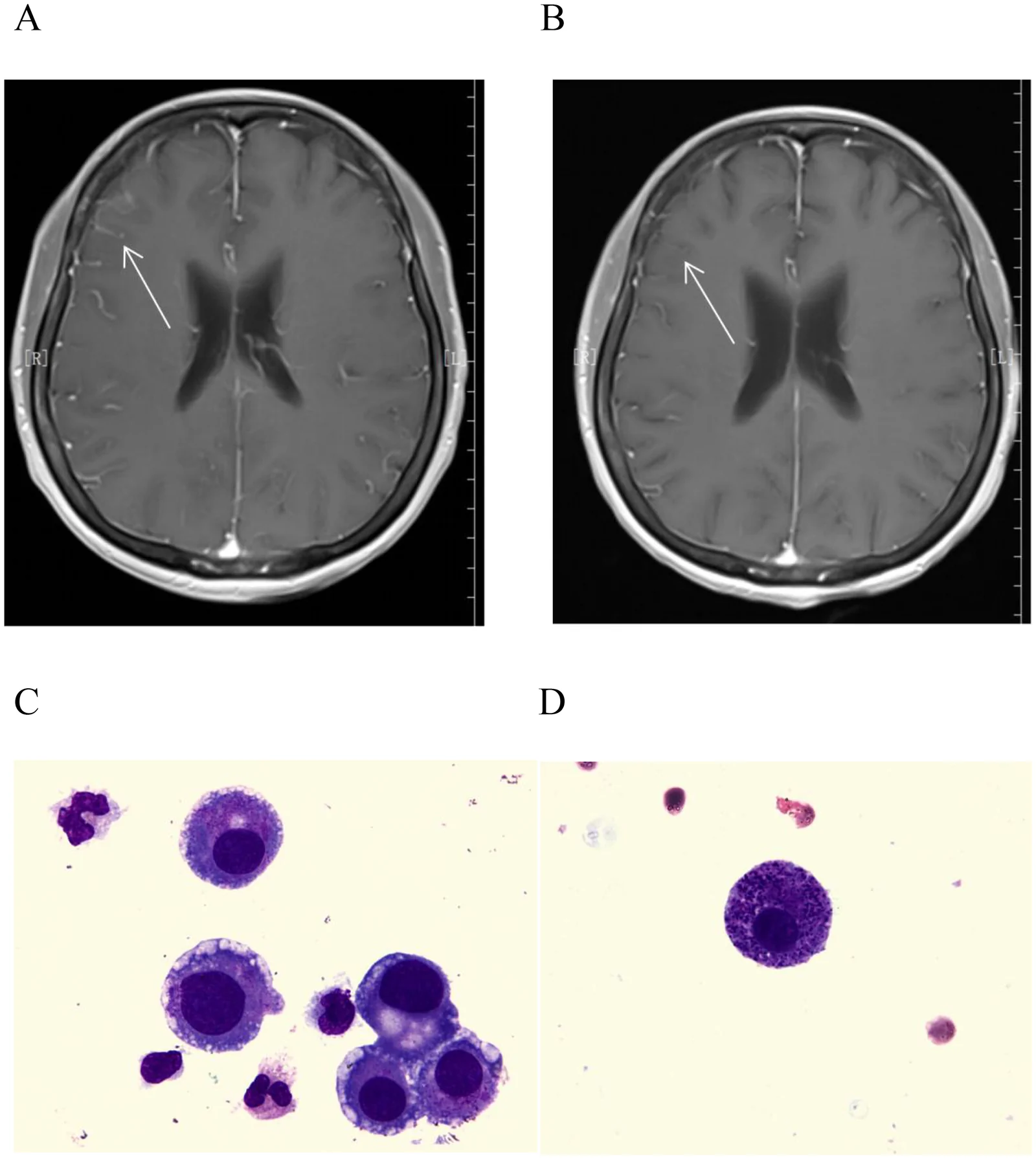
After treatment, MRI showed resolution of meningeal thickening and enhancement, and decreased tumor cells in CSF. MRI: T1-weighted contrast-enhanced, 5 mm slice thickness, scale bar = 1 cm. CSF cytology: Wright-Giemsa stain, ×1000. **(A)** Pre-treatment MRI. **(B)** Post-treatment MRI. **(C)** Pre-treatment CSF cytology. **(D)** Post-treatment CSF cytology.

## Case 3

A 47-year-old female was diagnosed with left-sided breast cancer in July 2018. Immunohistochemistry revealed ER-negative, PR-negative, HER2 3+, and Ki67 positivity of 50%, consistent with HER2-positive breast cancer. The patient’s initial diagnosis was stage IIIC (T2N3M0), with left axillary and supraclavicular lymph node metastases. The patient first received neoadjuvant therapy with epirubicin plus cyclophosphamide sequentially followed by docetaxel plus trastuzumab. On February 22, 2019, she underwent modified radical mastectomy of the left breast. The postoperative stage remained T2N3M0. Adjuvant therapy with trastuzumab plus pertuzumab was administered after surgery. In April 2020, she developed brain metastases involving the bilateral frontal lobes, left cerebellum, and right occipital lobe. During the advanced stage, the patient received two lines of antitumor therapy: pyrotinib plus nab-paclitaxel, and pyrotinib plus capecitabine.

In February 2023, the patient developed severe headache, dizziness, nausea, and vomiting. MRI revealed abnormal enhancing lesions in the meninges of the left cerebellar hemisphere and right occipital lobe, suggestive of LM. Lumbar puncture pathology demonstrated cancer cells in the CSF. In February 2023, the patient received intravenous T-DXd, combined with WBRT. After treatment, the patient’s central nervous system symptoms did not improve. However, the patient refused intrathecal injection, and her physical condition gradually deteriorated. The patient died on April 7, 2023. The patient’s PFS and OS were 2.2 months.

## Case 4

A 58-year-old female was diagnosed with left-sided breast cancer in December 2007. Immunohistochemistry revealed ER-positive, PR-positive, HER2 1+, and Ki67 positivity of 5%, consistent with luminal-type breast cancer. The patient’s initial diagnosis was stage IA (T1N0M0). On December 18, 2007, she underwent modified radical mastectomy of the left breast. In January 2010, she began to develop distant metastases involving the liver, chest wall, lymph nodes, and bones. During the advanced stage, the patient received eight lines of antitumor therapy: docetaxel plus capecitabine, goserelin plus letrozole, doxorubicin plus cyclophosphamide, gemcitabine plus cisplatin, palbociclib plus fulvestrant, eribulin, abemaciclib plus anastrozole, and vinorelbine.

In June 2023, the patient developed psychiatric symptoms and gait instability. MRI revealed thickening of the meninges in the bilateral cerebrum and cerebellum, with multiple cord-like and nodular enhancement, suggestive of LM (**Figure 2A**). Lumbar puncture pathology demonstrated cancer cells in the CSF. In June 2023, the patient received intravenous T-DXd plus bevacizumab, combined with WBRT, and weekly intrathecal injections of methotrexate plus cytarabine. After the initiation of intrathecal therapy, the patient’s central nervous system symptoms improved rapidly, and basically resolved after two intrathecal injections. Follow-up MRI after treatment revealed that the previous thickening and enhancement of the meninges in the bilateral cerebrum and cerebellum had decreased compared with before treatment (**Figure 2B**). In December 2024, the patient developed slow response and hearing loss. Follow-up MRI revealed enlargement of the meningeal enhancing lesions in the cerebellar vermis compared with previous scans, and the disease was assessed as progressive disease (PD). In addition, the patient developed malignant ascites and refused further antitumor therapy. The patient died on February 18, 2025. The patient’s PFS was 18.0 months, and OS was 20.2 months.

**Figure 2:**
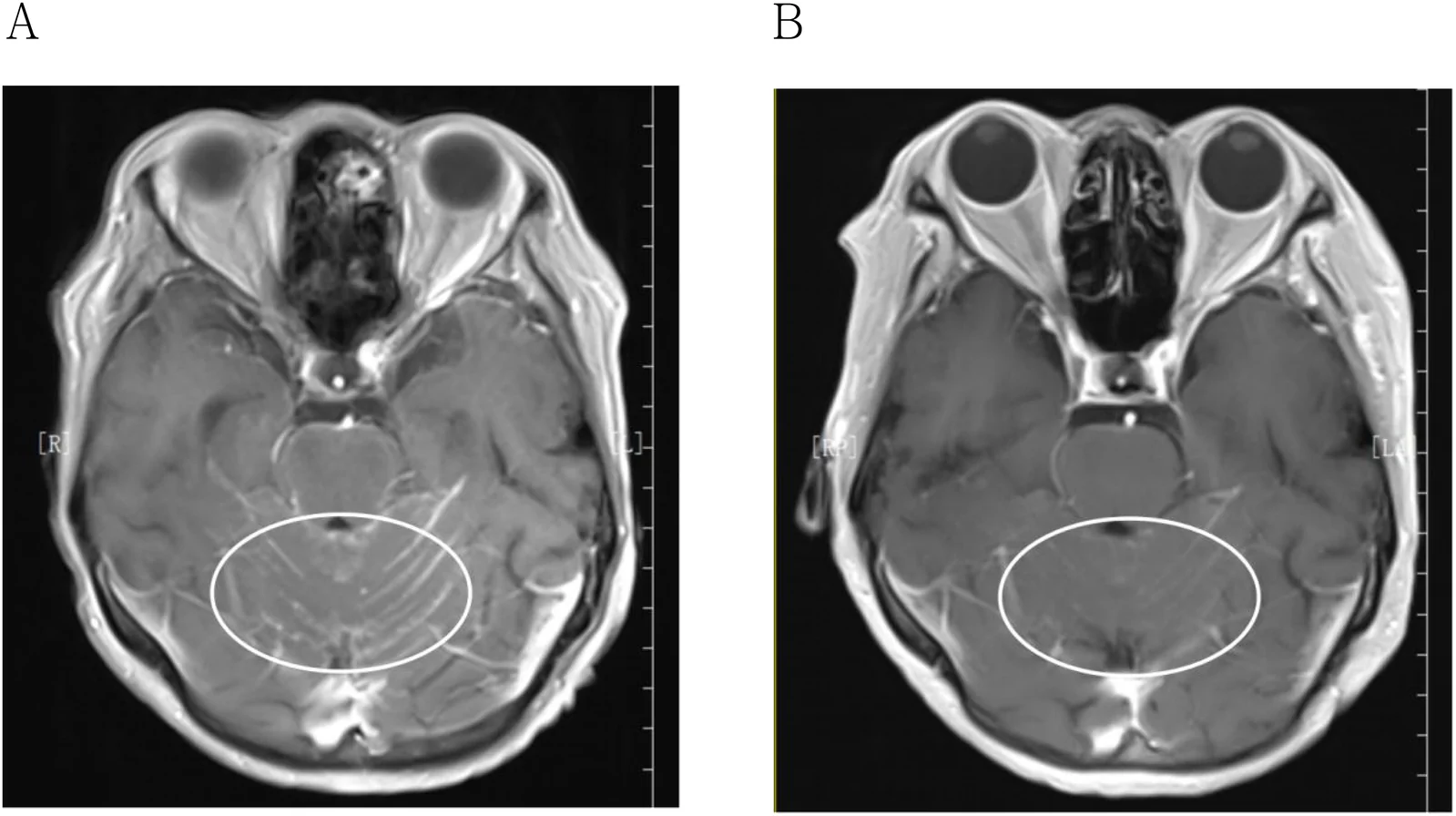
Post-treatment MRI showed reduced meningeal thickening and enhancement. **(A)** Pre-treatment MRI. **(B)** Post-treatment MRI.

## Case 5

A 56-year-old female was diagnosed with right-sided breast cancer in June 2022. Immunohistochemistry revealed ER-negative, PR-negative, HER2 (2+), and Ki67 positivity of 80%. Fluorescence *in situ* hybridization (FISH) testing for HER2 was negative, confirming TNBC. The patient’s initial diagnosis was stage IIIC (T1N3M0), with right axillary and supraclavicular lymph node metastases. Genetic testing revealed a germline *BRCA1* pathogenic mutation. The patient first received neoadjuvant therapy with docetaxel plus epirubicin plus cyclophosphamide. On October 11, 2022, she underwent modified radical mastectomy of the right breast. Adjuvant therapy with olaparib was administered after surgery.

In August 2023, the patient developed somnolence, confusion, and epileptic seizures. MRI revealed multiple marked enhancements in the bilateral sulci and meninges, with diffuse pial enhancement, suggestive of LM. Lumbar puncture biopsy demonstrated cancer cells in the CSF. On August 31, 2023, the patient received intravenous T-DXd, combined with WBRT. After treatment, the patient’s central nervous system symptoms did not improve. On September 2, 2023, the patient experienced a grand mal seizure, followed by secondary respiratory failure. The patient died on September 13, 2023. The patient’s PFS and OS were 0.8 months.

## Case 6

A 38-year-old female was diagnosed with right-sided breast cancer in June 2020. Immunohistochemistry revealed ER-positive, PR-positive, HER2 2+, and Ki67 positivity of 30%, consistent with luminal-type breast cancer. FISH testing for HER2 was negative. The patient’s initial diagnosis was stage IV (T4N3M1), with axillary lymph node, supraclavicular lymph node, and multiple bone metastases. Subsequently, she developed liver and spleen metastases. During the advanced stage, the patient received three lines of antitumor therapy: abemaciclib plus fulvestrant plus goserelin, docetaxel, and sacituzumab govitecan.

In August 2024, the patient developed psychiatric symptoms, dizziness, nausea, and vomiting. MRI revealed multiple linear enhancements in the sulci of the bilateral cerebrum and cerebellum, suggestive of LM (**Figures 3A, C**). Lumbar puncture pathology demonstrated cancer cells in the CSF. In August 2024, the patient received intravenous T-DXd plus bevacizumab, combined with WBRT, and weekly intrathecal injections of methotrexate plus cytarabine. After the initiation of intrathecal therapy, the patient’s central nervous system symptoms improved rapidly, and basically resolved after three intrathecal injections. Follow-up MRI after treatment revealed that the meningeal enhancing lesions in the bilateral cerebrum and cerebellum were significantly reduced compared with those before treatment (**Figures 3B, D**). In April 2025, the patient developed slow response, somnolence, and epileptic seizures. In addition, the patient developed ascites and jaundice, and refused further antitumor therapy. The patient died on June 4, 2025. The patient’s PFS was 8.2 months, and OS was 9.4 months.

**Figure 3:**
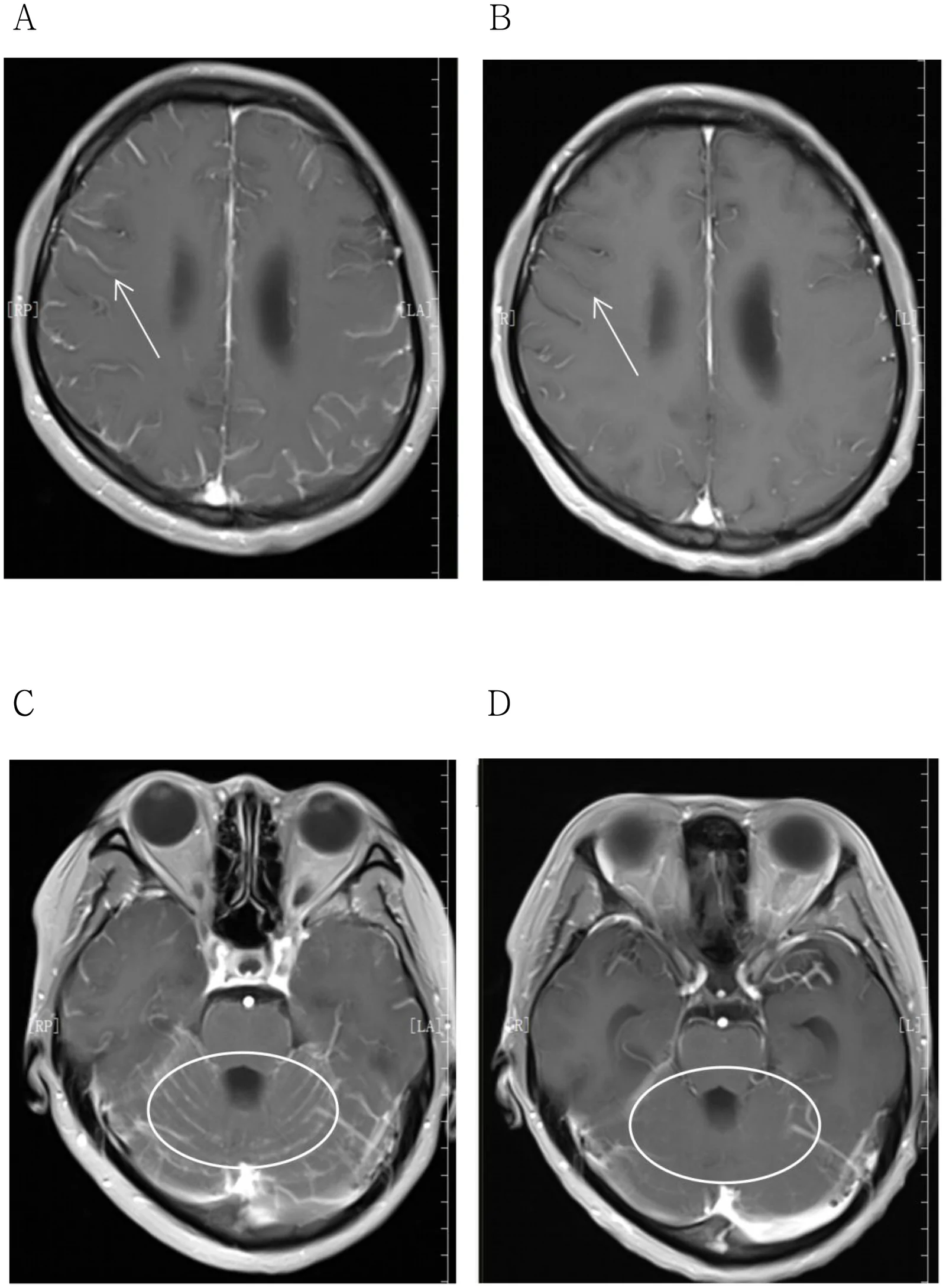
Post-treatment MRI showed markedly reduced meningeal enhancement. **(A, C)** Pre-treatment MRI. **(B, D)** Post-treatment MRI.

## Case 7

A 57-year-old female was diagnosed with left-sided breast cancer in September 2022. Immunohistochemistry revealed ER-negative, PR-negative, HER2 1+, and Ki67 positivity of 40%, consistent with TNBC. The patient’s initial diagnosis was stage IIIA (T2N2M0), with left axillary lymph node metastasis. The patient first received neoadjuvant chemotherapy with epirubicin plus cyclophosphamide sequentially followed by docetaxel plus carboplatin. In February 2023, she underwent modified radical mastectomy of the left breast. The postoperative stage remained T2N2M0. Adjuvant therapy with capecitabine was administered after surgery. In April 2024, she developed metastases to the supraclavicular and mediastinal lymph nodes. During the advanced stage, the patient received one line of antitumor therapy: pembrolizumab plus paclitaxel.

In February 2025, the patient developed hearing loss, tinnitus, and dizziness. MRI revealed nodular enhancing lesions in the right cerebellar hemisphere and vermis, suggestive of cerebellar metastases, without obvious meningeal thickening or enhancement. However, lumbar puncture pathology demonstrated cancer cells in the CSF. In February 2025, the patient received intravenous T-DXd plus bevacizumab, combined with WBRT, and weekly intrathecal injections of methotrexate plus cytarabine. After the initiation of intrathecal therapy, the patient’s tinnitus and dizziness improved rapidly, but hearing loss did not show significant recovery. In August 2025, the patient developed involuntary tremors of the head and both hands, with worsening of hearing loss compared with before. Follow-up MRI revealed that the metastatic lesions in the right cerebellar hemisphere and vermis had slightly enlarged compared with previous scans, with multiple abnormal pial enhancements observed. In August 2025, the treatment was switched to sacituzumab govitecan. In November 2025, the patient developed somnolence and confusion. The patient died on November 27, 2025. The patient’s PFS was 6.0 months, and OS was 9.0 months.

## Results

A total of seven patients were included. All patients received T-DXd plus WBRT; four also received bevacizumab and intrathecal injections. Patient characteristics, treatment details, and outcomes are summarized in **Table 1** and **Figure 4**. Intracranial best responses included partial response PR (n=3), stable disease (SD) (n=1), and PD (n=3). The median OS for the entire cohort was 9.0 months.

**Table 1:** Case Summary.

Patient number	Age (years)	Molecular subtyping	Genetic mutation	Presence of metastatic lesions	Central nervous system symptoms (Yes/No)	Main neurological symptoms	ECOG	Previous lines of therapy for advanced disease	T-DXd (Yes/No)	Bevacizumab (Yes/No)	Radiotherapy (Yes/No)	Intrathecal injection (Yes/No)	Best response	PFS (months)	OS (months)
1	76	HER2+	None	Liver, pericardium, pleura, chest wall, lymph nodes, bone	Yes	Dizziness, vomiting, gait instability, confusion	3	5	Yes	No	Yes	No	PD	2.1	2.1
2	48	TNBC	*BRCA2*	Ovary, pleura, bone	Yes	Severe headache, nausea, vomiting, blurred vision, epileptic seizures	3	2	Yes	Yes	Yes	Yes	PR	5.1	9.9
3	47	HER2+	None	Brain	Yes	Severe headache, dizziness, nausea, vomiting	3	2	Yes	No	Yes	No	PD	2.2	2.2
4	58	LuminalA	None	Liver, chest wall, lymph nodes, bone	Yes	Psychiatric symptoms, gait instability	1	8	Yes	Yes	Yes	Yes	PR	18.0	20.2
5	56	TNBC	*BRCA1*	None	Yes	Somnolence, confusion, epileptic seizures	4	0	Yes	No	Yes	No	PD	0.8	0.8
6	38	LuminalBHER2-	None	Liver, spleen, chest wall, bone	Yes	Psychiatric symptoms, dizziness, nausea, vomiting	2	3	Yes	Yes	Yes	Yes	PR	8.2	9.4
7	57	TNBC	None	Brain, lymph nodes	Yes	Hearing loss, tinnitus, dizziness	2	1	Yes	Yes	Yes	Yes	SD	6.0	9.0

*Best response was assessed for intracranial leptomeningeal lesions using the RANO-LM criteria. For patients who died before follow-up imaging, PD was based on clinical deterioration and physician assessment.

**Figure 4:**
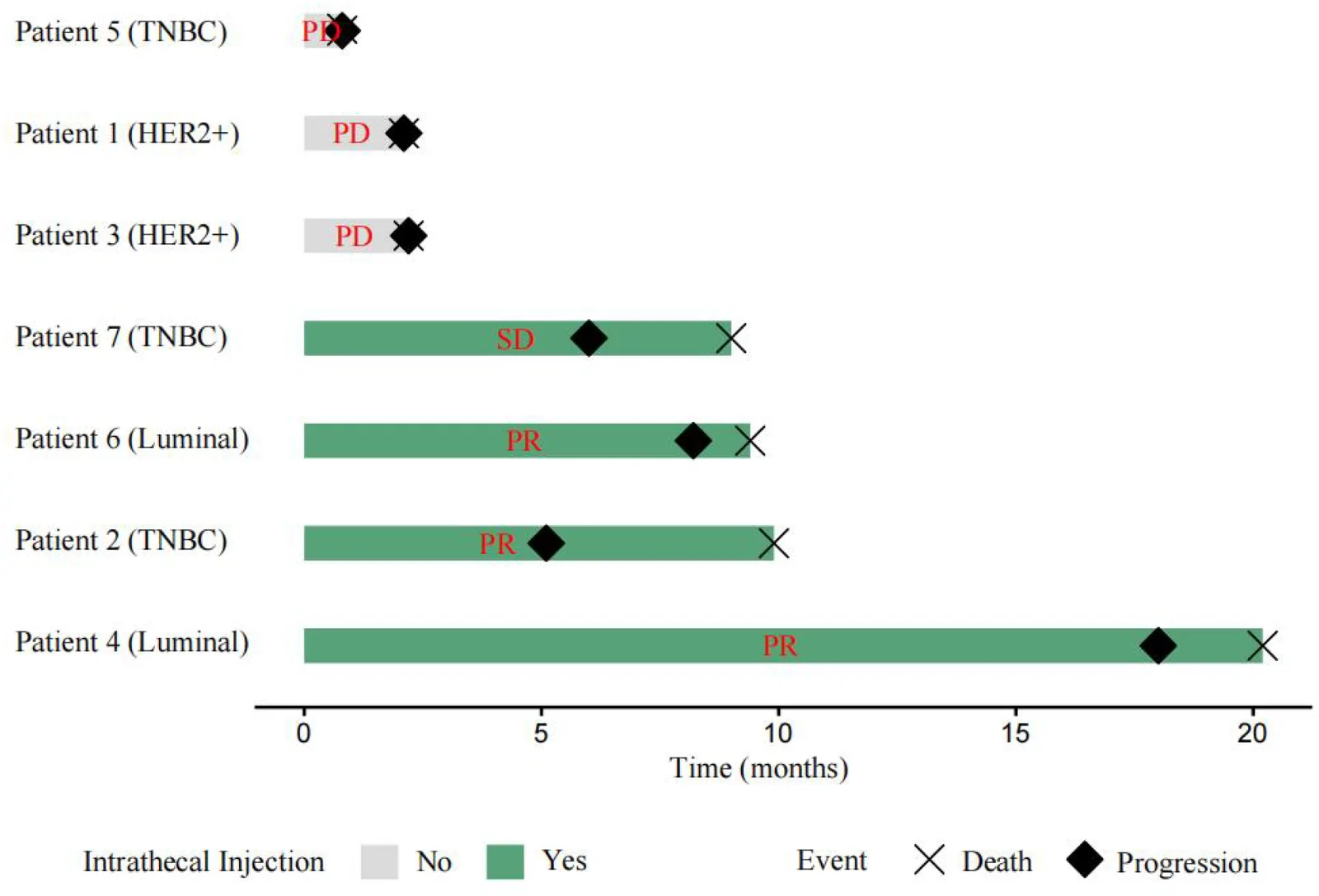
Swimmer plot illustrating the treatment timeline and survival duration for each patient from the start of LM-directed therapy to death or last follow-up.

The most common treatment-related adverse events were nausea, fatigue, and myelosuppression, predominantly grade 1–2. No cases of radiation necrosis or treatment-related deaths were reported. Intrathecal injections were well tolerated, with no chemical meningitis or severe headache requiring treatment interruption.

## Discussion

### Patient characteristics

This series included seven female patients with breast cancer LM, with a median age of 56 years (range 38–76). Molecular subtypes included HER2-positive (n=2), luminal A (n=1), luminal B HER2-negative (n=1), and TNBC (n=3). At the time of LM diagnosis, Eastern Cooperative Oncology Group (ECOG) performance status ranged from 1 to 4 (median 2), and all patients presented with CNS symptoms. LM was confirmed by CSF cytology in all cases. Prior to LM diagnosis, patients had received a median of 2 (range 0–8) prior lines of systemic therapy. Four patients received the quadruple regimen (T-DXd + WBRT + bevacizumab + intrathecal methotrexate/cytarabine), while three received T-DXd + WBRT alone. The median OS for the entire cohort was 9.0 months. Notably, the four patients who received the more intensive quadruple regimen achieved OS exceeding 9.0 months (range 9.0–20.2 months), whereas the three patients who did not receive bevacizumab or intrathecal therapy had OS of only 0.8–2.2 months.

### Diagnostic challenges

The incidence of leptomeningeal metastasis is low and its onset is insidious, making diagnosis challenging and prone to clinical missed diagnosis. According to the 2023 EANO-ESMO Clinical Practice Guideline for leptomeningeal metastases from solid tumors, the current diagnosis of LM mainly adopts a “diagnostic certainty” grading system, integrating clinical manifestations, imaging findings, and CSF cytology for comprehensive assessment (15). Patients presenting with central nervous system symptoms such as meningeal irritation signs or signs of intracranial hypertension should raise high suspicion of LM. Contrast-enhanced MRI is the preferred imaging modality, although some patients may show no obvious meningeal enhancement. CSF cytology is the “gold standard”; detection of malignant cells in the CSF confirms LM and enables timely initiation of targeted therapy. If cytology is negative but imaging shows typical findings, intervention should still be performed as highly suspected LM. If patients present with CNS symptoms but lack objective evidence, further examinations are required to confirm the diagnosis (16–18).

In this study, all seven patients presented with central nervous system symptoms, which drew our attention. All were diagnosed with LM via CSF cytology by lumbar puncture, and six of them showed typical meningeal thickening or enhancement on cranial MRI. However, in Case 7, the initial MRI only showed cerebellar metastases without obvious meningeal enhancement, while cancer cells were detected in the CSF. This reminds us that a negative MRI cannot exclude LM, and active lumbar puncture with CSF examination should be performed in patients with high clinical suspicion. This is particularly important for high-risk populations (patients with HER2-positive breast cancer, TNBC, or lobular carcinoma) who develop new cranial nerve symptoms, multifocal neurological signs, or unexplained encephalopathy. CSF circulating tumor DNA (ctDNA) testing is emerging as a powerful tool for the diagnosis and dynamic monitoring of LM, and may further improve diagnostic sensitivity and accuracy of treatment response assessment in the future (19). In addition, novel MRI techniques, such as diffusion-weighted imaging (DWI), fluid-attenuated inversion recovery (FLAIR), dynamic susceptibility contrast (DSC), and dynamic contrast-enhanced (DCE) sequences, can further improve the diagnostic rate of intracranial tumor lesions (20).

### Potential factors influencing prognosis

Although the overall prognosis of breast cancer LM is extremely poor, there are considerable differences in survival prognosis among individuals, influenced by multiple factors including patient characteristics, tumor features, and treatment modalities. This has prompted us to explore relevant prognostic factors and risk stratification tools. An analysis of 859 patients with LM from the German Brain Metastasis Registry (BMBC) showed that older age (≥60 years), poor performance status (ECOG 2–4), HR+/HER2− or triple-negative molecular subtypes, and a higher number of brain metastases were independent unfavorable prognostic factors (21). Multivariate analysis demonstrated that ECOG 2–4 was the independent risk factor with the worst prognosis, with a hazard ratio (HR) of 2.15, which was the highest HR among all variables included in the analysis, suggesting that performance status is the most critical prognostic indicator for survival in breast cancer patients with LM. In contrast, active antitumor treatment, including stereotactic radiotherapy, whole-brain radiotherapy, endocrine therapy, and anti-HER2 therapy, was significantly associated with longer survival. A multicenter study (NCT07386886) enrolled 403 breast cancer patients with LM, aiming to develop a novel graded prognostic assessment model specifically for breast cancer LM (BC-LGPA). This model is expected to become an important tool for future risk stratification, survival estimation, and clinical decision-making in LM.

In the limited cases of this study, Case 4 had the longest survival. Although this patient had previously received multiple lines of antitumor therapy, her molecular subtype was luminal A-type, with relatively indolent biological behavior, and her ECOG performance status was the best among all cases in this study. In addition, the patient received more aggressive combined systemic and local antitumor therapy. Cases 1, 3, and 5 had very short survival, with OS of 2.1, 2.2, and 0.8 months, respectively. Case 1 was older in age. Case 3 had an ECOG performance status of 3 at the time of LM diagnosis. Case 5 had TNBC and an ECOG performance status of 4. The above clinical characteristics are consistent with the unfavorable prognostic factors for LM (21). Meanwhile, the common feature of these three patients was that they did not receive more intensive antitumor therapy, including intrathecal injection and bevacizumab. This suggests that although the overall prognosis of breast cancer LM is poor, different treatment strategies can have a substantial impact on patient survival. For LM patients with acceptable performance status, active antitumor treatment should be administered to prolong survival.

### Survival benefit of the quadruple-combination treatment regimen

Due to the blood–brain barrier, drug therapy for intracranial tumors, especially LM, faces challenges and bottlenecks. For HER2-positive breast cancer LM, current studies have shown that TKIs (such as tucatinib) and ADCs (such as T-DXd) exhibit promising intracranial activity, with intracranial mPFS exceeding 6 months (22, 23). In the DEBBRAH study (cohort 5), which included seven patients with LM, T-DXd achieved a mOS of 13.3 months and a mPFS of 8.9 months, with a clinical benefit rate (CBR, defined as stable disease for ≥24 weeks) of 71.4% (24). A real-world study (ROSET-BM) further confirmed the efficacy of T-DXd in LM, with a mPFS of 17.5 months in 19 patients with LM (25). Traditionally, large-molecule antibody drugs were considered unable to penetrate the blood–brain barrier, limiting their application in central nervous system (CNS) metastases. However, the clinical activity of T-DXd in LM challenges this notion. Preclinical studies have revealed that T-DXd can cross the blood–tumor barrier via FcRn-mediated transcytosis to enter the CNS (26). Once inside the CNS, the HER2 antibody component of T-DXd specifically binds to tumor cells, and the cleavable linker releases the highly membrane-permeable DXd payload, which kills surrounding tumor cells through the bystander effect. Combination with radiotherapy can further disrupt the blood–brain barrier, enhance ADC delivery, and exert synergistic effects. In a study of T-DXd combined with stereotactic radiosurgery (SRS) for HER2-positive breast cancer brain metastases, radiotherapy combined with T-DXd demonstrated promising intracranial tumor control rates (27). In that study, 13% of patients had LM, and the 12-month CNS local control rate reached 97%. Radiotherapy is a very important treatment modality for LM; however, previous studies have shown that T-DM1 combined with radiotherapy significantly increases the risk of radiation necrosis (28). This may be related to the fact that the microtubule inhibitor payload DM1 of T-DM1 binds to and damages reactive astrocytes activated after radiotherapy, thereby exacerbating radiation injury to brain tissue (29). Current studies have shown that the rate of radiation necrosis with T-DXd combined with SRS is extremely low, suggesting that the safety profile of different ADCs combined with radiotherapy may differ significantly (27). No radiation necrosis was observed in any of the seven patients with breast cancer LM who received T-DXd combined with WBRT. However, overall survival varied considerably among patients (range 0.8–20.2 months), suggesting room for further optimization of the current treatment strategy. The heterogeneity in treatment response may be related to molecular subtype, baseline performance status, prior lines of therapy, and differential sensitivity to various treatment components.

Several clinical studies have observed the value of bevacizumab in intracranial metastases from breast cancer. A prospective phase II study enrolled patients with brain metastases from solid tumors who had progressed after whole-brain radiotherapy and had limited treatment options, of whom 78% had metastatic breast cancer, with a median of six prior lines of antitumor therapy before enrollment. Salvage therapy with the addition of bevacizumab to existing systemic treatment showed promising survival benefits in patients with brain metastases who had lost standard treatment options, with a mPFS of 5.3 months and a mOS of 9.5 months (30). A real-world study included MBC patients with brain metastases who received bevacizumab-containing combination regimens. The intracranial objective response rate (ORR) reached 74.0%, and could be further improved to 81.2% when combined with radiotherapy (31). In a retrospective study of non-small cell lung cancer patients with LM, patients received intrathecal pemetrexed combined with systemic therapy, of whom 83.8% received bevacizumab. Multivariate analysis showed that the combination of bevacizumab was significantly associated with longer survival (32). Currently, a prospective study of bevacizumab combined with chemotherapy for breast cancer patients with brain metastases or LM is ongoing (NCT01281696). Bevacizumab can improve the delivery of drugs to the central nervous system through vascular normalization, creating favorable conditions for the synergistic effects of intrathecal chemotherapy and systemic T-DXd. In a phase II study, bevacizumab combined with chemotherapy was administered to breast cancer patients with brain metastases or LM (14). The results showed that the plasma concentration of bevacizumab was positively correlated with overall survival, and higher concentrations were associated with greater ability of chemotherapeutic agents to penetrate the blood–brain barrier. The dosage of bevacizumab varies across different tumors; in this study, the dose was 15 mg/kg administered intravenously every three weeks. In our cohort, given the generally poor performance status of LM patients and the intensity of the multimodal regimen, we opted for a reduced dose of 7.5 mg/kg every three weeks. With this dose, the overall safety profile was favorable, and no hypertension or hemorrhagic events were observed in our patients. Future prospective studies with larger sample sizes should be conducted to validate the efficacy of bevacizumab in breast cancer patients with intracranial metastases, and to explore the optimal regimen and dosage.

Intrathecal injection is an effective means of bypassing the blood–brain barrier and directly increasing drug concentrations in the cerebrospinal fluid, and has accumulated considerable clinical research experience in the treatment of breast cancer LM. The most commonly used chemotherapeutic agents are methotrexate and cytarabine, which have favorable safety profiles for intrathecal administration; therefore, both were used in this study. An international multicenter phase I/II study enrolled 26 patients with HER2-positive LM, who received intrathecal trastuzumab at a dose of 80 mg twice weekly. The mOS of LM patients receiving intrathecal trastuzumab was 10.5 months, with generally good tolerability; one patient developed grade 4 arachnoiditis (33). Another phase II study enrolled 19 HER2-positive breast cancer patients with LM and evaluated the efficacy of intrathecal trastuzumab at a dose of 150 mg once weekly. In that study, the LM-related mPFS was 5.9 months, and mOS was 7.9 months. Patients’ overall quality of life was maintained or improved, and central nervous system symptoms were alleviated. After treatment, three grade 3 toxicities were observed, including seizure, headache, and allergic reaction (34). Currently, Fudan University Shanghai Cancer Center is conducting a phase I/II clinical trial (INOVATE study, NCT07134153) of intrathecal/Ommaya reservoir-administered T-DXd in HER2-expressing breast cancer patients with LM. This study will address whether intrathecal T-DXd can overcome the blood–brain barrier limitation and improve central drug exposure. In our cohort, no severe adverse events such as meningitis or seizure were observed during intrathecal therapy. However, given the potential risks associated with repeated lumbar punctures, we elected to discontinue intrathecal injections after CNS symptoms had resolved. This approach was taken to avoid unnecessary procedural complications, although it remains unclear whether a longer course of intrathecal therapy might have provided additional tumor control. Drawing an analogy to the treatment paradigm in hematological malignancies — where induction and consolidation phases employ different intrathecal dosing frequencies — a similar risk-adapted strategy might also be considered in LM to achieve better disease control. Nevertheless, the optimal frequency, dosage, and regimen of intrathecal therapy for LM still require further investigation.

In this study, all seven patients received T-DXd combined with WBRT, among whom four additionally received intravenous bevacizumab and intrathecal injections of methotrexate plus cytarabine (i.e., quadruple-combination therapy). The OS of these four patients was 9.9, 20.2, 9.4, and 9.0 months, respectively, all exceeding 9.0 months, with three achieving PR and one achieving SD. In contrast, the three patients who did not receive bevacizumab or intrathecal injections had OS of 2.1, 2.2, and 0.8 months, respectively, and all were assessed as having PD. Although statistical comparison could not be performed due to the small sample size, the numerical difference in survival time between the two groups was considerable. This suggests that, on the basis of systemic T-DXd therapy and whole-brain radiotherapy, the addition of bevacizumab and intrathecal chemotherapy may further improve survival outcomes in patients with LM.

Notably, beyond the survival advantage, patients in the quadruple-combination group also experienced meaningful symptomatic relief. From the perspective of patients’ quality of life, all four patients who received intrathecal injections combined with bevacizumab reported rapid improvement in their central nervous system symptoms. In Case 2, severe headache resolved after three intrathecal injections, allowing the patient to discontinue opioid analgesics and dehydrating agents; no further epileptic seizures were observed during subsequent follow-up. In Case 4, psychiatric symptoms and gait instability improved after two intrathecal injections, and the patient was able to resume independent ambulation. In Case 6, dizziness and vomiting resolved after three intrathecal injections. In Case 7, tinnitus and dizziness improved; although hearing loss persisted, the patient expressed satisfaction with the relief of dizziness. These patient-reported improvements in overall well-being suggest that, in addition to potential survival benefits, the addition of intrathecal therapy and bevacizumab may provide meaningful symptomatic relief and quality of life enhancement.

### Study limitations and future directions

This study has several limitations that should be considered when interpreting our findings.

First, this was a retrospective, non-randomized study, which inherently carries a risk of selection bias. Patients who received the quadruple regimen had better baseline performance status compared with those who did not, which may have contributed to their longer survival independently of treatment. Therefore, we cannot conclude that the quadruple regimen is definitively superior based on these data alone. Nonetheless, the magnitude of the survival difference — 9.0–20.2 months in the quadruple group versus 0.8–2.2 months in the control group — is considerable, and the quadruple group included patients with aggressive subtypes (e.g., triple-negative breast cancer) who would not be expected to have exceptionally favorable outcomes. This numerical difference, while not statistically testable, suggests that the regimen warrants further investigation.

Second, the small sample size (n=7) precludes multivariate adjustment or reliable subgroup analyses. Consequently, our findings are best viewed as hypothesis-generating rather than confirmatory. Nevertheless, even small case series can provide valuable clinical signals, particularly in rare conditions such as LM where prospective data are scarce.

Third, the concurrent use of multiple treatment modalities prevents us from disentangling the individual contribution of each component. We acknowledge this as a major limitation of our study. However, from a clinical perspective, LM is a complex condition that often requires multimodal management; our case series reflects real-world practice where combination therapy is frequently used.

Fourth, the lack of standardized neurocognitive function and quality of life assessments limits our ability to evaluate the impact of this intensive regimen on patients’ functional status. Nevertheless, the rapid symptomatic improvement reported by patients provides some indirect evidence that the regimen may offer meaningful clinical benefit beyond survival prolongation.

Future prospective studies with systematic adverse event collection are needed. Future research directions should include: first, conducting prospective, randomized controlled phase II studies to further evaluate the safety and efficacy of the quadruple-combination regimen. Second, exploring the feasibility of novel intrathecal agents, such as liposomal cytarabine, trastuzumab, and even intrathecal administration of T-DXd, to improve antitumor efficacy. Third, attempting to utilize CSF ctDNA for dynamic monitoring, aiming to more accurately assess treatment response, identify resistance early, and guide treatment adjustments.

## Conclusion

This case series suggests that the quadruple-combination regimen consisting of T-DXd-based systemic therapy combined with whole-brain radiotherapy, bevacizumab, and intrathecal injections shows potential survival benefits with an acceptable safety profile in breast cancer patients with LM. Particularly for patients with prominent central nervous system symptoms, intrathecal injections combined with bevacizumab can rapidly alleviate symptoms and improve quality of life. Due to the limited sample size, these findings require further validation in prospective studies. For patients with highly aggressive LM, this comprehensive treatment model may represent a potential clinical option.

## Data Availability

The original contributions presented in the study are included in the article/supplementary material. Further inquiries can be directed to the corresponding author.
